# Supplementary material for: Conditional quantum operation of two exchange-coupled single-donor spin qubits in a MOS-compatible silicon device
Source: Nat Commun. 2021 Jan 8;12:181. doi: 10.1038/s41467-020-20424-5 (PMC7794236; doi:10.1038/s41467-020-20424-5)
Supplement: Supplementary file 1 — Supplementary Information [file 41467_2020_20424_MOESM1_ESM.pdf]

SUPPLEMENTARY INFORMATION: Conditional quantum operation of two exchange-coupled single-donor spin qubits in a MOS-compatible silicon device

Mateusz T. Mądzik[1], Arne Laucht[1], Fay E. Hudson[1], Alexander M. Jakob[2], Brett C. Johnson[2], David N. Jamieson[2], Kohei M. Itoh[3], Andrew S. Dzurak[1], and Andrea Morello[1*]

1. Centre for Quantum Computation and Communication Technology, School of Electrical Engineering and Telecommunications, UNSW Sydney, Sydney NSW 2052, Australia

2. Centre for Quantum Computation and Communication Technology, School of Physics, University of Melbourne, Melbourne VIC 3010, Australia and

3. School of Fundamental Science and Technology, Keio University, 3-14-1 Hiyoshi, 223-8522, Japan

I. SUPPLEMENTARY NOTE 1 – QUANTUM DESCRIPTION OF AN EXCHANGE-COUPLED ^{31}P DONOR PAIR

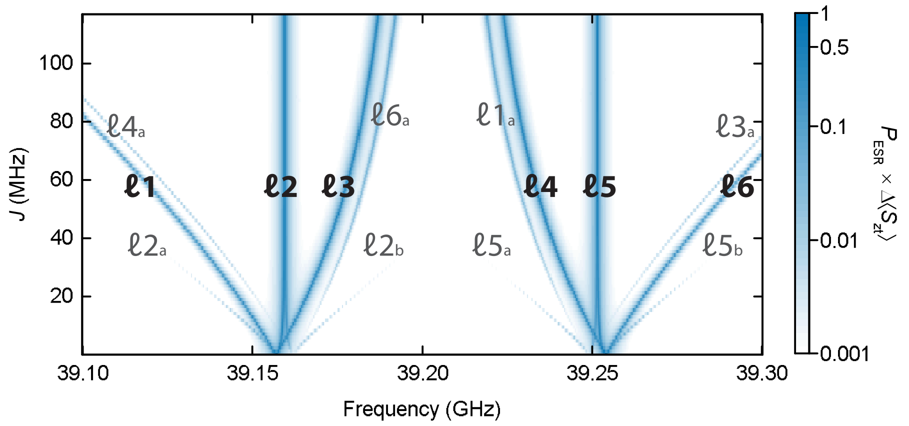

Supplementary Figure 1 | Electron spin resonance spectrum of weakly exchange-coupled ^{31}P electron spin qubits. Simulated electron spin resonance (ESR) spectrum of a donor pair, as a function of exchange coupling J, using the two-donor Hamiltonian in Eq. 1.

In this section we provide the background theory of exchange-coupled donor spin qubits, necessary to understand their behaviour when subjected to the resonant microwave excitations that can constitute a two-qubit CROT gate. The spectroscopic study of exchange-coupled donor spins has a long history[1], but here we focus on an embodiment of two-qubit operations that requires local, individual control of all electron and nuclear spins in the donor pair[2].

We consider two coupled ^{31}P donor spin qubits in silicon, in which one acts as the 'target' (subscript t) and the other as the 'control' (subscript c) in a two-qubit quantum logic operation. The spin Hamiltonian (in frequency units) of the donors placed in a static magnetic field B_0 (≈ 1.4 T in our experiment) is described by the electron ($\mathbf{S}_\mathrm{t}, \mathbf{S}_\mathrm{c}$, with basis states $|\uparrow\rangle, |\downarrow\rangle$) and nuclear ($\mathbf{I}_\mathrm{t}, \mathbf{I}_\mathrm{c}$, with basis states $|\Uparrow\rangle, |\Downarrow\rangle$) spin 1/2 vector Pauli operators. In the presence of a Heisenberg exchange coupling J, the Hamiltonian takes the form:

$$H = (\mu_\mathrm{B}/h)B_0(g_\mathrm{t}S_{z\mathrm{t}} + g_\mathrm{c}S_{z\mathrm{c}}) + \gamma_n B_0(I_{z\mathrm{t}} + I_{z\mathrm{c}}) + A_\mathrm{t}\mathbf{S}_\mathrm{t}\cdot\mathbf{I}_\mathrm{t} + A_\mathrm{c}\mathbf{S}_\mathrm{c}\cdot\mathbf{I}_\mathrm{c} + J(\mathbf{S}_\mathrm{t}\cdot\mathbf{S}_\mathrm{c}), \quad (1)$$

where μ_B is the Bohr magneton, h is the Planck constant, and $g_\mathrm{t}, g_\mathrm{c} \approx 1.9985$ are the Landé g-factors, such that $g\mu_\mathrm{B}/h \approx 27.97$ GHz/T. The nuclear gyromagnetic ratio is $\gamma_n \approx -17.23$ MHz/T, and $A_\mathrm{t}, A_\mathrm{c}$ are the electron-nuclear contact hyperfine interactions in the target and in the control donor, respectively; their average is $\bar{A} = (A_\mathrm{t} + A_\mathrm{c})/2$ and their difference $\Delta A = (A_\mathrm{t} - A_\mathrm{c})$. In bulk donors $A = 117.53$ MHz, but in a nanoscale electronic device each atom may have a different A due to local wavefunction distortions induced by strain and/or electric fields[3,4].

With this Hamiltonian, we can calculate the outcome of an ESR experiment where an oscillating magnetic field $B_1\cos(2\pi\nu t)$ applied along the x-direction induces transitions between an initial and final eigenstate $|\psi_\mathrm{i}\rangle, |\psi_\mathrm{f}\rangle$, with probability $P_\mathrm{ESR} = |\langle\psi_\mathrm{i}|(\sigma_{x\mathrm{c}} + \sigma_{x\mathrm{t}})|\psi_\mathrm{f}\rangle|^2$. In the main text, we presented an experiment where the excitations are detected by reading out the z-projection of the target qubit. Therefore, in the simulation we multiply P_ESR of each transition by the change in expectation value of that qubit between initial and final state, i.e. by $\Delta\langle S_{z\mathrm{t}}\rangle = |\langle\psi_\mathrm{f}|S_{z\mathrm{t}}|\psi_\mathrm{f}\rangle - \langle\psi_\mathrm{i}|S_{z\mathrm{t}}|\psi_\mathrm{i}\rangle|$. The complete ESR spectrum, calculated over all possible initial electron and nuclear eigenstates, is shown in Supplementary Figure 1. It exhibits six main resonance lines, labelled $\boldsymbol{\ell}1\ldots\boldsymbol{\ell}6$, plus eight faint resonances ($\boldsymbol{\ell}1a\ldots\boldsymbol{\ell}6a$, $\boldsymbol{\ell}2b$, $\boldsymbol{\ell}5b$).

To understand the features of this ESR spectrum, we first consider the parameter range of relevance for a CROT gate, namely $|\Delta A| \ll J \ll \bar{A}$. If the nuclear spins are in a parallel orientation, $|\Downarrow_\mathrm{c}\Downarrow_\mathrm{t}\rangle$ or $|\Uparrow_\mathrm{c}\Uparrow_\mathrm{t}\rangle$, the eigenstates of the system are the tensor products of the nuclear states with the electron singlet, $|S\rangle = (|\uparrow_\mathrm{c}\downarrow_\mathrm{t}\rangle - |\downarrow_\mathrm{c}\uparrow_\mathrm{t}\rangle)/\sqrt{2}$, and triplet states, $|T_-\rangle = |\downarrow_\mathrm{c}\downarrow_\mathrm{t}\rangle$, $|T_0\rangle = (|\uparrow_\mathrm{c}\downarrow_\mathrm{t}\rangle + |\downarrow_\mathrm{c}\uparrow_\mathrm{t}\rangle)/\sqrt{2}$, $|T_+\rangle = |\uparrow_\mathrm{c}\uparrow_\mathrm{t}\rangle$. The corresponding ESR lines are $\boldsymbol{\ell}2$ (active when the nuclei are in the state $|\Downarrow_\mathrm{c}\Downarrow_\mathrm{t}\rangle$) and $\boldsymbol{\ell}5$ ($|\Uparrow_\mathrm{c}\Uparrow_\mathrm{t}\rangle$). Each of these lines is doubly degenerate, since it describes transitions between $|T_-\rangle \leftrightarrow |T_0\rangle$ and $|T_0\rangle \leftrightarrow |T_+\rangle$ that have identical frequencies. Because of this degeneracy, the excitation of one spin does not depend on the state of the other.

A conditional 2-qubit operation becomes possible if the nuclei are prepared in opposite state. In this case, the $\widetilde{\text{Hamiltonian}}$ eigenstates are the tensor products of the nuclear states ($|\Downarrow_\mathrm{c}\Uparrow_\mathrm{t}\rangle$ or $|\Uparrow_\mathrm{c}\Downarrow_\mathrm{t}\rangle$) with the electronic states $|\downarrow_\mathrm{c}\downarrow_\mathrm{t}\rangle$, $\widetilde{|\uparrow_\mathrm{c}\downarrow_\mathrm{t}\rangle}$, $\widetilde{|\downarrow_\mathrm{c}\uparrow_\mathrm{t}\rangle}$, $|\uparrow_\mathrm{c}\uparrow_\mathrm{t}\rangle$, where $\widetilde{|\uparrow_\mathrm{c}\downarrow_\mathrm{t}\rangle} = \cos\theta\,|\uparrow_\mathrm{c}\downarrow_\mathrm{t}\rangle + \sin\theta\,|\downarrow_\mathrm{c}\uparrow_\mathrm{t}\rangle$, $\widetilde{|\downarrow_\mathrm{c}\uparrow_\mathrm{t}\rangle} = \cos\theta\,|\downarrow_\mathrm{c}\uparrow_\mathrm{t}\rangle - \sin\theta\,|\uparrow_\mathrm{c}\downarrow_\mathrm{t}\rangle$, and $\tan(2\theta) = J/\bar{A}$.

This situation is equivalent to that found in double quantum dot systems, but here the energy detuning $\delta\epsilon$ between the qubits is provided by the hyperfine coupling $\bar{A}$ instead of a field gradient[5] or a g-factor difference[6].

The corresponding ESR lines come in pairs characterized by a common nuclear state. For $|{\Downarrow_c}{\Uparrow_t}\rangle$ we find $\boldsymbol{\ell}1$, describing the transition $|{\downarrow_c}{\downarrow_t}\rangle \leftrightarrow \widetilde{|{\downarrow_c}{\uparrow_t}\rangle}$, and $\boldsymbol{\ell}3$ ($\widetilde{|{\uparrow_c}{\downarrow_t}\rangle} \leftrightarrow |{\uparrow_c}{\uparrow_t}\rangle$), while for $|{\Uparrow_c}{\Downarrow_t}\rangle$ the same electronic transitions are represented by $\boldsymbol{\ell}4$ and $\boldsymbol{\ell}6$, respectively (see Supplementary Figure 1). The frequency separation between $\boldsymbol{\ell}1$ and $\boldsymbol{\ell}3$, and between $\boldsymbol{\ell}4$ and $\boldsymbol{\ell}6$, is precisely the exchange coupling J. Since the frequencies of these four transitions depend on the state of the control qubit, a selective π-pulse on each transition represents a two-qubit conditional gate operation, as depicted in the Fig. 2**b** of the main text.

Under the condition of $|\Delta A| \ll J \ll \bar{A}$, the resonance frequencies corresponding to the six main ESR lines $\boldsymbol{\ell}1 \ldots \boldsymbol{\ell}6$ are expressed by:

$$
\begin{aligned}
\boldsymbol{\ell}1 =& \gamma_e B_0 + \frac{\Delta A}{2} - \frac{J}{2} - \frac{\sqrt{\overline{A}^2 + J^2}}{2} \\
\boldsymbol{\ell}2 =& \gamma_e B_0 - \frac{\overline{A}}{2} \\
\boldsymbol{\ell}3 =& \gamma_e B_0 + \frac{\Delta A}{2} + \frac{J}{2} - \frac{\sqrt{\overline{A}^2 + J^2}}{2} \\
\boldsymbol{\ell}4 =& \gamma_e B_0 + \frac{\Delta A}{2} - \frac{J}{2} + \frac{\sqrt{\overline{A}^2 + J^2}}{2} \\
\boldsymbol{\ell}5 =& \gamma_e B_0 + \frac{\overline{A}}{2} \\
\boldsymbol{\ell}6 =& \gamma_e B_0 + \frac{\Delta A}{2} + \frac{J}{2} + \frac{\sqrt{\overline{A}^2 + J^2}}{2}
\end{aligned}
\qquad (2)
$$

The simulated ESR spectrum in Supplementary Fig. 1 highlights several additional faints resonances: $\boldsymbol{\ell}1_a \ldots \boldsymbol{\ell}6_a$, $\boldsymbol{\ell}2_b$, $\boldsymbol{\ell}5_b$. These resonances appear for antiparallel nuclear configurations because, when $J > 0$, the eigenstates of the two-electron system include the partially entangled $\widetilde{|{\uparrow_c}{\downarrow_t}\rangle}$, $\widetilde{|{\downarrow_c}{\uparrow_t}\rangle}$. This has some important consequences. Firstly, consider e.g. the transition from $|{\downarrow_c}{\downarrow_t}\rangle$ to $\widetilde{|{\downarrow_c}{\uparrow_t}\rangle}$. Although the most probable outcome is flipping the target electron (as intended in a CROT gate), there is a probability $\sin^2\theta$ of flipping the control electron, with possible repercussions on the subsequent operations. Secondly, a transition addressing the control electron can be visible also while observing only the target qubit. Consider for instance $\boldsymbol{\ell}4_a$ and $\boldsymbol{\ell}6_a$: They are the "sister resonances" (i.e. with the same nuclear spin orientation, $|{\Downarrow_c}{\Uparrow_t}\rangle$) of lines $\boldsymbol{\ell}4$ and $\boldsymbol{\ell}6$ for the target electron, but they are detected at the frequencies corresponding to $\boldsymbol{\ell}1$ and $\boldsymbol{\ell}3$ for the control electron. These lines appear and increase in intensity once $J > |\Delta A|$.

If J is increased further, beyond the value of $\bar{A}$, the eigenstates of the Hamiltonian (1) evolve into singlet and triplet electron states. ESR transitions associated with what becomes the singlet state ($\boldsymbol{\ell}2_a$, $\boldsymbol{\ell}1$, $\boldsymbol{\ell}4_a$, $\boldsymbol{\ell}2_b$, $\boldsymbol{\ell}5_a$, $\boldsymbol{\ell}3_a$, $\boldsymbol{\ell}6$, $\boldsymbol{\ell}5_b$) will progressively vanish as J increases, since the singlet has total spin $S = 0$ and constitutes an ESR inactive state. Spin transitions can be induced between electron triplet states, but they all have the same frequency, independent of J. Therefore, the branches $\boldsymbol{\ell}3$, $\boldsymbol{\ell}6_a$, $\boldsymbol{\ell}1_a$ and $\boldsymbol{\ell}4$ merge into a single line located between the trivial resonances ($\boldsymbol{\ell}2$ and $\boldsymbol{\ell}5$). The regime $J > \bar{A}$ is of no interest for the implementation of resonant CROT gates (the corresponding ESR spectrum is thus not shown in Supplementary Fig. 1), but can become the basis for a native SWAP gate[2].

II. SUPPLEMENTARY NOTE 2 – DONOR IMPLANTATION STRATEGIES

Two different implantation strategies have been considered in this experiment, a single ion P^+ implantation and a P_2^+ molecule implantation. The key difference between these two methods is the post-implantation inter-donor separation. Supplementary Figure 2**a** shows the predicted dependence of the P-P distance on the implantation fluence, for the two implantation methods. For fluences larger than $\approx 3 \times 10^{11}/\mathrm{cm}^2$ the two methods yield the same P-P distance. This is because donors originating from one P_2^+ molecule have a high likelihood of coming to rest close to donors coming from other P_2^+ molecules: this results in the same spatial distribution that would be obtained by randomly placing individual ions at the same density. At low fluences the P_2^+ molecular implantation yields an average P-P distance that saturates to a constant dependent only on the implantation energy, since that it the parameter that sets how far apart the two atoms in each molecule are likely to come to rest.

Supplementary Figure 2**b** shows a comparison of P-P distance histograms calculated assuming P^+ and P_2^+ implantation strategies at the same fluence of 5×10^{10} atoms/cm^{-2} and the same energy per atom (10 keV). At this low fluence, the P_2^+ molecular ions yield a higher likelihood of finding closely spaces pairs. This dose was selected for the device whose charge stability diagram is depicted in Figure 2**a** of the main text. Nonetheless, our experiments showed that this implantation strategies is not sufficient to give enough yield, and was thus replaced with a high fluence P^+ implantation at 1.25×10^{12} ions/cm^2.

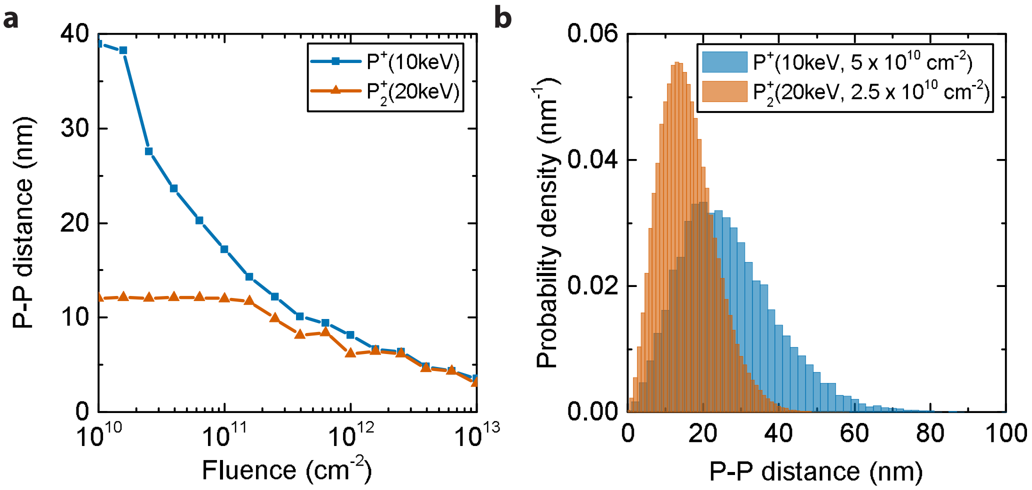

Supplementary Figure 2 | Comparison of ion (P^+) and molecular (P_2^+) implantation strategies. Blue represents P^+ ions, and orange P_2^+ molecules. **a**, Inter-donor spacing as a function of implantation fluence. **b**, Comparison of P-P distance probability density for the two implantations strategies with the same total number of atoms, and same energy per atom.

III. SUPPLEMENTARY NOTE 3 – SINGLE-QUBIT PROPERTIES OF THE TARGET ELECTRON

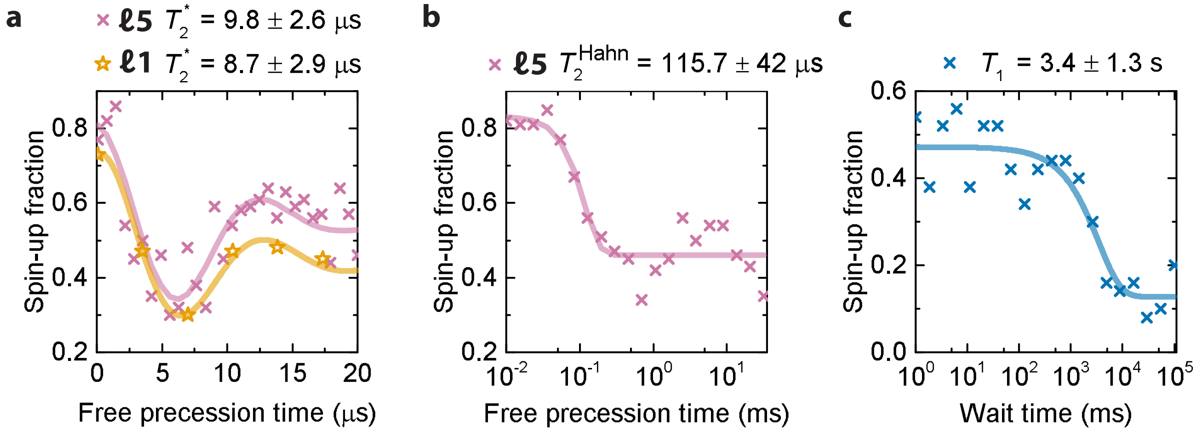

Supplementary Figure 3 | Single qubit properties of the target electron measured in the (1_c,1_t) charge state. a, Pure dephasing time extracted from a Ramsey experiment, with $T_2^* = 9.8 \pm 2.6$ μs measured on the unconditional $\ell5$ resonance and $T_2^* = 8.7 \pm 2.9$ μs measured on the conditional $\ell1$ resonance. **b**, Hahn echo coherence time $T_2^{\mathrm{Hahn}} = 115 \pm 42$ μs measured on the ESR line $\ell5$. **c**, Longitudinal relaxation time T_1 of the target qubit, obtained by randomly loading a $|\downarrow\rangle$ or $|\uparrow\rangle$ state on the donor and measuring the time decay of the $|\uparrow\rangle$ probability. In a static external magnetic field $B_0 = 1.4$ T, we found $T_1 = 3.4 \pm 1.3$ s. All error bars indicate 95% confidence levels.

Key properties of the target qubit have been characterized in the presence of exchange coupling, by measuring Ramsey fringes, Hahn echo and longitudinal relaxation while tuning the two-donor system in the (1_c, 1_t) charge

configuration.

In Supplementary Figure 3**a** we show measurements of pure dephasing time T_2^* of the target electron, on an unconditional resonance ($\boldsymbol{\ell}5$, pink), and on a conditional resonance ($\boldsymbol{\ell}1$, orange). Within the experimental error, both resonances yield the same dephasing time, with an extracted $T_2^* = 8.7 \pm 2.9$ μs on $\boldsymbol{\ell}1$, and $T_2^* = 9.8 \pm 2.6$ μs on $\boldsymbol{\ell}5$. This is an important result, which provides preliminary confirmation that the use of weak exchange coupling does not significantly affect the dephasing time of the qubits.

The Hahn echo coherence time was measured as $T_2^{\mathrm{Hahn}} = 115 \pm 42$ μs on ESR line $\boldsymbol{\ell}5$ (Supplementary Figure 3**b**).

The T_1 measurement of the target electron was conducted with the use of random loading at 1.4 T. The target donor is ionized by pulsing to the charge sector (1_c, 0_t), and then pulsing back into the (1_c,1_t) to load an electron in a random spin state. A variable wait time is introduced before reading out the electron spin state. This method results in $\approx 50\%$ probability of loading $|{\downarrow_\mathrm{c}\uparrow_\mathrm{t}}\rangle$ at the start of the wait time, and allows for a T_1 measurement without ESR control. The relaxation time for the target qubit was $T_1 = 3.4 \pm 1.3$ s. This value is comparable to that of single, uncoupled ^{31}P electron spin qubits[7].

IV. SUPPLEMENTARY NOTE 4 – SINGLE-SHOT READOUT TRACES FROM RABI OSCILLATIONS EXPERIMENTS

As shown in Fig. 5 of the main text, there is a clear difference in the visibility of the Rabi oscillations between the conditional and unconditional transitions. One of the possible explanations for this effect is that reading out the target qubit may project the two-electron system from the initial $\widetilde{|{\downarrow_\mathrm{c}\uparrow_\mathrm{t}}\rangle} = 0.986\,|{\downarrow_\mathrm{c}\uparrow_\mathrm{t}}\rangle + 0.166\,|{\uparrow_\mathrm{c}\downarrow_\mathrm{t}}\rangle$ state into the $|{\uparrow_\mathrm{c}\downarrow_\mathrm{t}}\rangle$, whereby the control qubit is flipped to the $|{\uparrow}\rangle$ state. This would render the $\boldsymbol{\ell}1$ and $\boldsymbol{\ell}4$ inactive for the T_1 time of the control electron. A signature of this effect should be clearly visible in the raw traces of the experiment. We extracted the raw SET current data traces obtained after applying a π-pulse on each of the resonances $\boldsymbol{\ell}1$, $\boldsymbol{\ell}2$, $\boldsymbol{\ell}4$, $\boldsymbol{\ell}5$, and plot them in Supplementary Figure 4. Each panel shows a total of 60 consecutive single-shot readouts of the target electron. In a perfect experiment, every readout shot would have a SET current spike, indicating successful rotation of the target spin.

For the conditional ESR resonances ($\boldsymbol{\ell}1$ and $\boldsymbol{\ell}4$), we have marked the regions where it is plausible that the possible control electron might have flipped to $|{\uparrow}\rangle$: these are identified by the absence of $|{\uparrow}\rangle$ signal on the target qubit, occurring on multiple consecutive shots. While such instances exist, they don't seem to persist for more than 4-5 consecutive shots at a maximum. Therefore, for this to be caused by the projection to $|{\uparrow_\mathrm{c}\downarrow_\mathrm{t}}\rangle$ upon readout, we would have to assume a control electron $T_1 \approx 20$ ms. This is an unlikely scenario, since T_1 of the target electron has been measured to be two orders of magnitude longer.

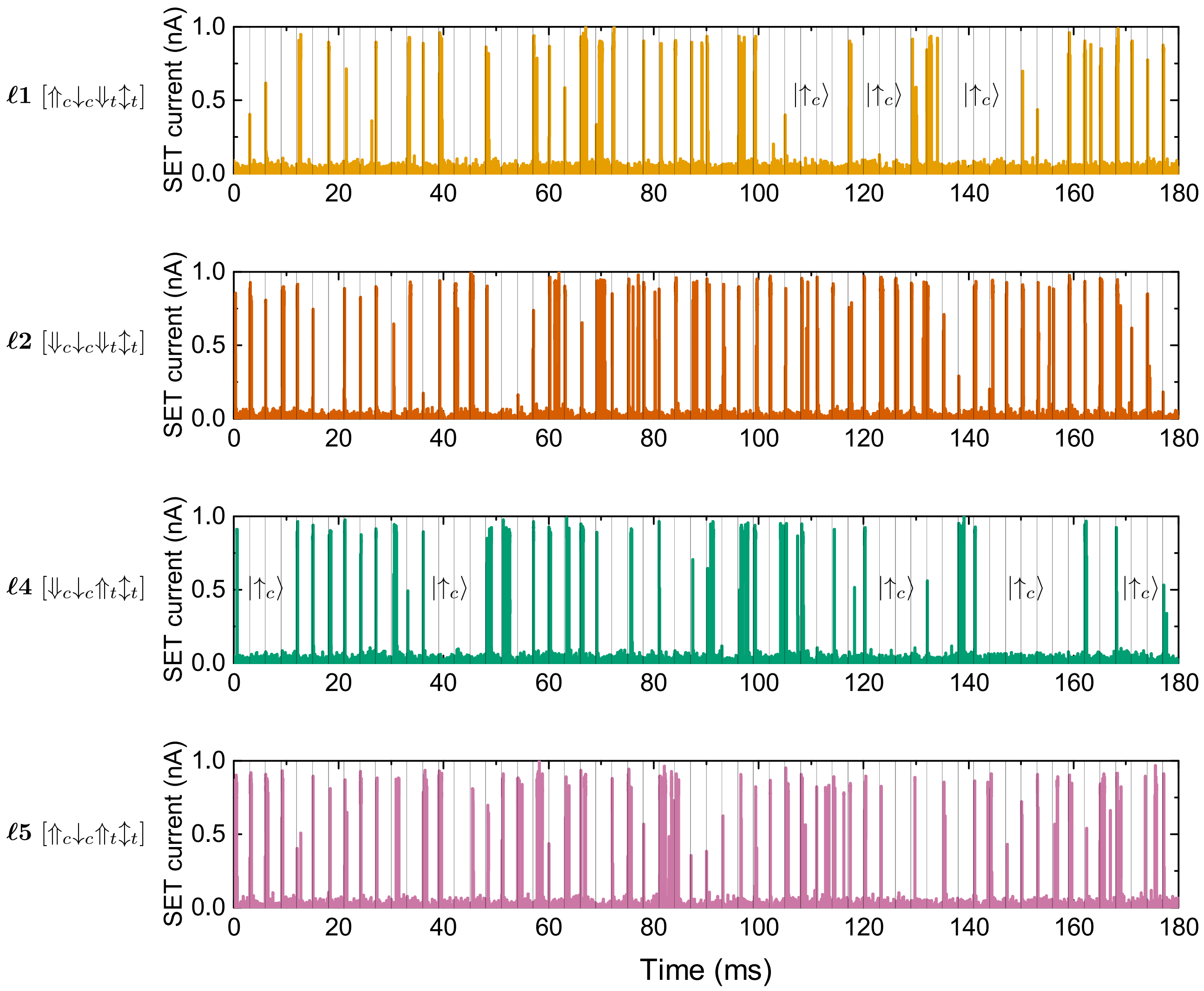

Supplementary Figure 4 | Raw traces of SET current after a π-pulse on each resonance. The traces correspond to the π rotation data point in the Rabi experiment summarized in Figure 4 of the main text. Each electron spin readout window is 3 ms long. A high-current blip indicates the ionization of the target donor, and used to identify the $|\uparrow\rangle$ state[8].

V. SUPPLEMENTARY NOTE 5 – NUCLEAR SPIN INITIALIZATION

Nuclear spin initialization is performed before the manipulation of the electron spin qubits. The extremely long relaxation time of the nuclear spins, governed mostly by the electron ionization events[9], allows for a ”set and forget” approach.

The Supplementary Figure 5 summarizes the nuclear spin initialization procedure. Although the electron two-qubit control must be performed in the $(1_c, 1_t)$ charge configuration (orange star), the nuclear initialization is best executed in $(1_c, 0_t)$ charge state (blue star). This is because, in the (unlikely, but not implausible) case where both target and control qubits in the coupled donor pair have identical hyperfine couplings, the NMR frequencies of the two nuclei would overlap while operating in the $(1_c, 1_t)$ charge configuration (see Fig. 5**b**). Conversely, in the $(1_c, 0_t)$ charge configuration we remove the hyperfine coupling term for the target qubit nucleus, thus detuning the two nuclear resonance frequencies by $A/2$. This ensures that we can individually address each nucleus.

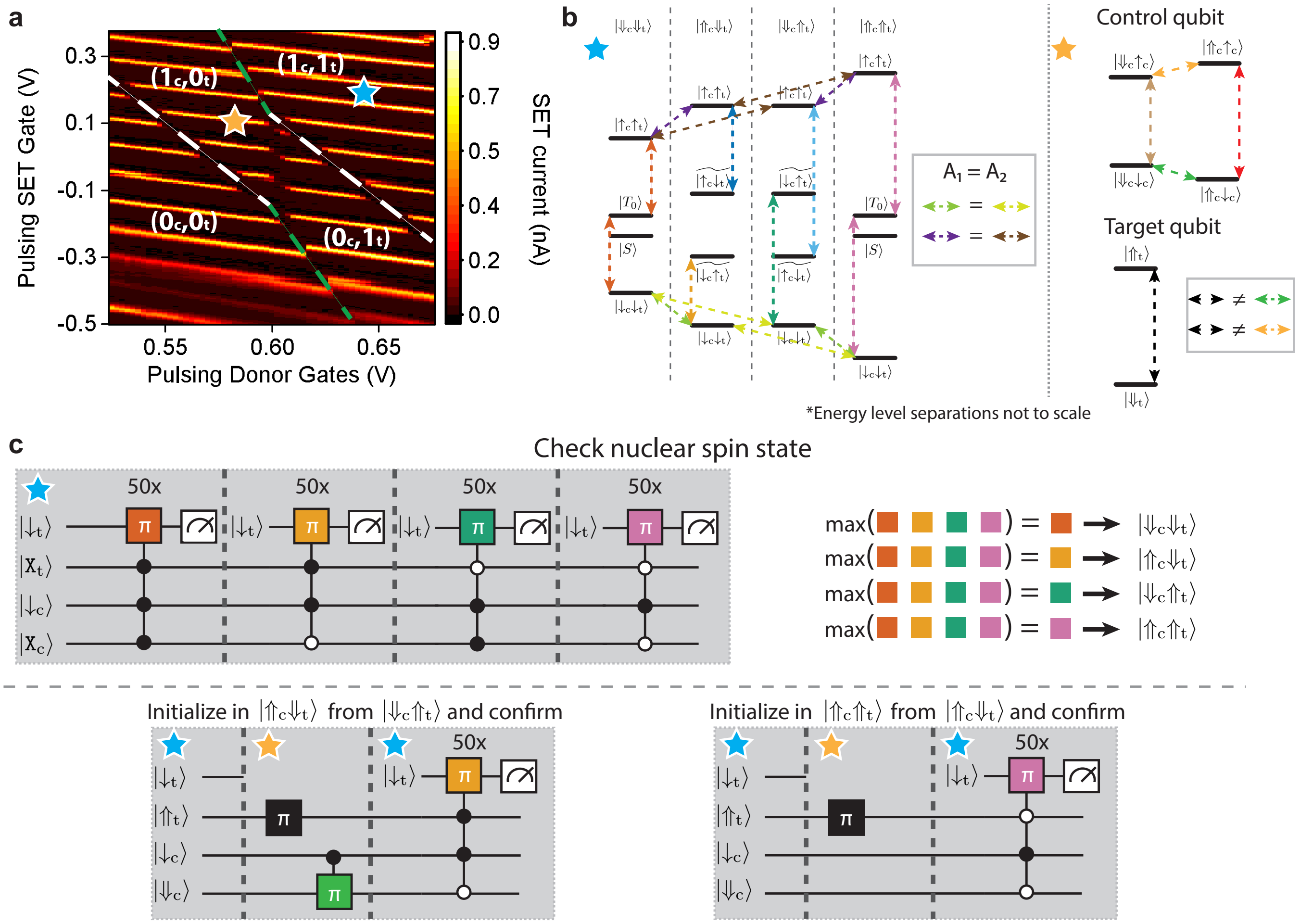

Supplementary Figure 5 | Nuclear spin initialization. **a,** Charge stability diagram around two donor charge transitions, obtained by scanning the voltages on the pulsing SET and the pulsing donor gates. **b,** [LEFT] Electronic energy level diagram of a pair of ^{31}P donors in the $(1_c, 1_t)$ charge configuration; we assume here for simplicity $A_c = A_t = A$ and $J \ll A$. With $\Delta A = A_c - A_t = 0$, the NMR frequencies for both nuclei would be equal, preventing individual control. [RIGHT] Electronic energy level diagram of a pair of ^{31}P donors in $(1_c, 0_t)$ charge configuration. With one donor ionized, the two Phosphorus atoms behave as individual, decoupled systems. **c,** The nuclear spin initialization starts with probing each of the four nuclear spin configurations via conditional ESR pulses in the $(1_c, 1_t)$. By comparing the measured electron spin-up probabilities corresponding to each nuclear spin configuration, we can deduce the current nuclear spin state. Subsequently, we can ionize one of the donors to achieve individual addressing of the nuclei and apply NMR pulses to reach any desired nuclear spin state. Two examples of nuclear spin initialization are included in the figure.

VI. SUPPLEMENTARY NOTE 6 – OBSERVATION OF EXCHANGE COUPLING IN A SECOND DEVICE

We present here the ESR spectrum of a second device, fabricated using the high-fluence P^+ implantation strategy. This device, like the one described in the main text, included an on-chip microwave antenna terminated by a very thin short-circuit, with a $\approx 50 \times 50$ nm^2 cross-section. Such antenna design turned out to be prone to damage by electrostatic discharges, as discussed extensively in Ref.[10]. In that paper, the damaged termination turned the antenna into an open circuit at NMR frequencies, and enabled the discovery of Nuclear Electric Resonance of a single ^{123}Sb nucleus, through the electrical modulation of nuclear quadrupole interaction.

Here, however, we deal with ^{31}P nuclei which possess no nuclear quadrupole. Therefore, once the antenna termination is damaged, it is no longer possible to perform NMR, since the antenna produces no oscillating magnetic field at frequencies of tens of MHz. However, the impedance of damaged antenna remains small enough at 40 GHz to allow some degree of ESR control[10].

The Supplementary Figure 6 shows the ESR spectrum of a second device, acquired using the adiabatic frequency sweep as in Figure 4 of the main text. Because of the inability to perform NMR, we simply repeated the measurements many times and waited for a spontaneous flip of the nuclear spins. We succeeded in identifying lines $\ell 1$, $\ell 2$, $\ell 3$ and $\ell 5$. The distance between $\ell 1$ and $\ell 3$ indicated an exchange coupling $J \approx 30$ MHz, remarkably similar to that of the device described in the main text.

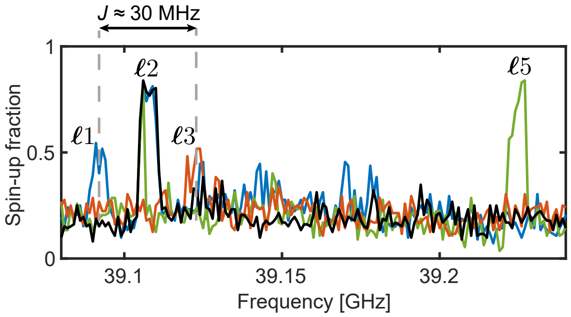

Supplementary Figure 6 | ESR spectrum and exchange coupling in a second device. A second device, fabricated with high-fluence P^+ implantation, shows evidence of $\approx 30 \pm 1$ MHz exchange coupling. The ESR spectrum is artificially broadened by the use of adiabatic inversion.

---

* a.morello@unsw.edu.au

[1] Cullis, P. R. & Marko, J. Determination of the donor pair exchange energy in phosphorus-doped silicon. *Physical Review B* **1**, 632 (1970).
[2] Kalra, R., Laucht, A., Hill, C. D. & Morello, A. Robust two-qubit gates for donors in silicon controlled by hyperfine interactions. *Physical Review X* **4**, 021044 (2014).
[3] Mansir, J. *et al.* Linear hyperfine tuning of donor spins in silicon using hydrostatic strain. *Phys. Rev. Lett.* **120**, 167701 (2018).
[4] Laucht, A. *et al.* Electrically controlling single-spin qubits in a continuous microwave field. *Science Advances* **1**, e1500022 (2015).
[5] Zajac, D. M. *et al.* Resonantly driven CNOT gate for electron spins. *Science* **359**, 439–442 (2018).
[6] Huang, W. *et al.* Fidelity benchmarks for two-qubit gates in silicon. *Nature* **569**, 532 (2019).
[7] Tenberg, S. B. *et al.* Electron spin relaxation of single phosphorus donors in metal-oxide-semiconductor nanoscale devices. *Physical Review B* **99**, 205306 (2019).
[8] Pla, J. J. *et al.* A single-atom electron spin qubit in silicon. *Nature* **489**, 541 (2012).
[9] Pla, J. J. *et al.* High-fidelity readout and control of a nuclear spin qubit in silicon. *Nature* **496**, 334 (2013).
[10] Asaad, S. *et al.* Coherent electrical control of a single high-spin nucleus in silicon. *Nature* **579**, 205–209 (2020).
