# Supplementary material for: Conditional quantum operation of two exchange-coupled single-donor spin qubits in a MOS-compatible silicon device
Source: Nat Commun. 2021 Jan 8;12:181. doi: 10.1038/s41467-020-20424-5 (PMC7794236; doi:10.1038/s41467-020-20424-5)
Supplement: Supplementary file 3 — Description of Additional Supplementary Files [file 41467_2020_20424_MOESM3_ESM.pdf]

Description of Additional Supplementary Files

Title: Supplementary Movie 1

Description: Evolution of the charge stability diagrams (SET current vs. SET top gate and right donor gate) while sweeping the left donor gate. The charge transition corresponding to the target qubit (diagonal break in the pattern of SET current peaks) shifts from higher to lower right-donor gate voltages upon increasing the left donor gate voltage, crossing through the control qubit charge transition. This enables us to choose which qubit is read out first.
