# Supplementary figures and images for: Conditional quantum operation of two exchange-coupled single-donor spin qubits in a MOS-compatible silicon device
Source: Nat Commun. 2021 Jan 8;12:181. doi: 10.1038/s41467-020-20424-5 (PMC7794236; doi:10.1038/s41467-020-20424-5)

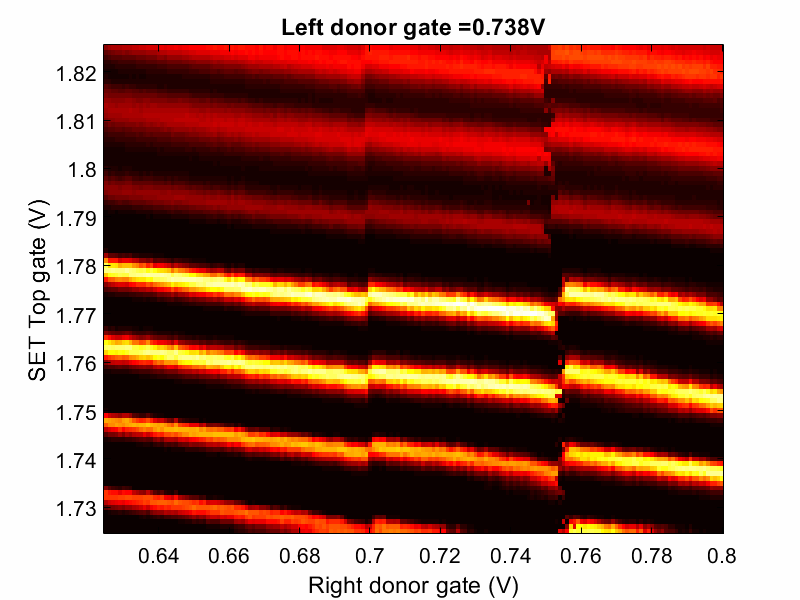

Supplement: Supplementary file 4 — Supplementary Movie 1 [file 41467_2020_20424_MOESM4_ESM.gif]
